# Notoginsenoside R1 Regulates Ischemic Myocardial Lipid Metabolism by Activating the AKT/mTOR Signaling Pathway

**DOI:** 10.3389/fphar.2022.905092

**Published:** 2022-06-22

**Authors:** Wei Lei, Yiqi Yan, Yaolei Ma, Min Jiang, Boli Zhang, Han Zhang, Yuhong Li

**Affiliations:** ^1^ Key Laboratory of Pharmacology of Traditional Chinese Medical Formulae, Ministry of Education, Tianjin University of Traditional Chinese Medicine, Tianjin, China; ^2^ State Key Laboratory of Component-based Chinese Medicine, Tianjin University of Traditional Chinese Medicine, Tianjin, China; ^3^ Institute of Traditional Chinese Medicine, Tianjin University of Traditional Chinese Medicine, Tianjin, China; ^4^ State Key Laboratory of Medicinal Chemical Biology, College of Pharmacy and Tianjin Key Laboratory of Molecular Drug Research, Nankai University, Tianjin, China

**Keywords:** notoginsenoside R1, ischemic heart disease, cardio-protection, lipid metabolism, Akt/mTOR pathway

## Abstract

Ischemic heart diseases are responsible for more than one-third of all deaths worldwide. *Radix notoginseng* is widely used to treat ischemic heart disease in China and other Asian countries, and notoginsenoside R1 (NGR1) is its characteristic and large-amount ingredient. However, the potential molecular mechanisms of NGR1 in improving ischemic heart diseases are unclear. In this study, we combined pharmacological evaluation with network pharmacology, myocardial proteomics, and conventional molecular dynamics (MD) simulation to explore the cardio-protection mechanisms of NGR1. Our results revealed that NGR1 improved the echocardiographic, tissue pathological, and serum biochemical perturbations in myocardial ischemic rats. The network pharmacology studies indicated that NGR1 mainly regulated smooth muscle cell proliferation, vasculature development, and lipid metabolism signaling, especially in the PI3K/AKT pathway. Myocardial proteomics revealed that the function of NGR1 was focused on regulating metabolic and energy supply processes. The research combined reverse-docked targets with differential proteins and demonstrated that NGR1 modulated lipid metabolism in ischemic myocardia by interacting with mTOR and AKT. Conventional MD simulation was applied to investigate the influence of NGR1 on the structural stabilization of the mTOR and AKT complex. The results suggested that NGR1 can strengthen the affinity stabilization of mTOR and AKT. Our study first revealed that NGR1 enhanced the affinity stabilization of mTOR and AKT, thus promoting the activation of the AKT/mTOR pathway and improving lipid metabolic abnormity in myocardial ischemic rats.

## 1 Introduction

Every year, ischemic heart diseases are responsible for more than one-third of all deaths worldwide, and this percent has been continuously increasing (Lei et al., 2021). Myocardial ischemia, which is characterized by a sharp decline in oxygen and nutrient supply for cardiomyocytes, results in a shift of the energy supply mode and the accumulation of lipids, especially fatty acids, in cardiomyocytes (Du et al., 2018). The accumulation of too many lipids becomes one of the vital causes of ischemia-induced arrhythmia, systolic dysfunction, myocardial infarction, and heart failure (Feng et al., 2018). Therefore, timely restoration of normal energy supply and alleviation of the accumulation of lipids are a promising therapeutic strategy for ischemic heart diseases.

*Radix notoginseng* (also called *Sanchi*), the dried root of Panax notoginseng (Burk.) F.H. Chen, is widely used as a functional food or dietary supplement in the United States and Asian and European countries (Zhang et al., 2018). During the centuries of usage of *Radix notoginseng*, this functional food exerts obvious benefits of enhancing blood circulation, cleaning up blood stasis, relieving pain, and remitting swelling (Chan et al., 2019). Thus, *Radix notoginseng* is adopted for the prevention and treatment of ischemic heart disease, diabetes mellitus, and cerebrovascular disease in China and other Asian countries (Guo et al., 2019). Notoginsenoside R1 (NGR1), a dammarane-type saponin, is a characteristic and large-amount component in *Radix notoginseng* and is defined as the quality control marker for this herb in Chinese pharmacopeia (Xu et al., 2018). Although many pharmacological functions, such as anti-inflammation, antioxidation, and prevention of myocardial fibrosis, had been reported to be responsible for the improvement of NGR1 to ischemic heart disease, there have been few research studies concerning the lipid metabolic regulation effect of NGR1 on ischemic heart disease.

Therefore, our study attempts to investigate the lipid metabolic regulation effect of NGR1 on ischemic heart diseases and discusses the potential mechanisms of NGR1 regulating lipid metabolism. The acute myocardial ischemia (MI) model was established by ligating the left anterior descending coronary artery (LADCA) of rats, and the therapeutic effects of NGR1 were evaluated. Network pharmacology combined with myocardial proteomics was performed to determine the lipid metabolic regulation effect of NGR1 on MI rats and identify the potential targets of NGR1. Guiding by the reverse-docked targets of NGR1 and differentially expressed proteins, the pivotal mechanisms of NGR1 regulating lipid metabolism in ischemic myocardium were discussed by conventional molecular dynamics (MD) simulations. The present study eventually clarified that NGR1 regulated lipid metabolism in the ischemic myocardium by enhancing the binding affinity of mTOR and AKT, thus ameliorating ischemic heart diseases.

## 2 Materials and Methods

### 2.1 Ethics

All animal procedures rigorously complied with the National Institutes of Health Guide for the Care and Use of Laboratory Animals (NIH publications No. 8023, revised 1978), and these animal experiments were approved by the Tianjin University of Traditional Chinese Medicine of Laboratory Animal Care and Use Committee with the ethical permission number of TCM-LAEC2020052.

### 2.2 Chemicals and Reagents

Notoginsenoside R1 (NGR1) (PubChem CID: 441934) was purchased from Tianjin Solomon Bio-technology Co., Ltd. (Tianjin, China). Metoprolol tartrate (Met) was bought from AstraZeneca Pharmaceutical Co., Ltd. (Shanghai, China). The primary antibodies against cyclooxygenase-2 (COX2) (Cat# ab179800), carnitine O-palmitoyltransferase 2 (CPT2) (Cat# ab110293), and platelet glycoprotein 4 (CD36) (Cat# ab252922) were obtained from Abcam (Cambridge, MA, United States). The primary antibodies against very long-chain specific acyl-CoA dehydrogenase (ACADVL) (Cat# PB1076) and glyceraldehyde-3-phosphate dehydrogenase (GAPDH) (Cat# BA2913) were purchased from Boster Biological Technology Co. Ltd. (Wuhan, China). The HRP-conjugated secondary antibodies (Cat# bs-40295G-HRP) were bought from Bioss, Inc. (Beijing, China). The lactate dehydrogenase (LDH) kit, creatine kinase-MB (CK-MB) kit, α-hydroxybutyrate dehydrogenase (α-HBDH) kit, free-fatty acid (FFA) kit, and enzyme-linked immunosorbent assay (ELISA) kits for rat interleukin-6 (IL-6), nuclear factor kappa-B (NF-κB), and tumor necrosis factor-alpha (TNF-α) were purchased from Jiancheng Bioengineering Institute (Nanjing, China). All other chemicals and solvents applied were of analytical grade.

### 2.3 Animals

Male Sprague–Dawley rats, from the SiPeiFu Laboratory Animal Technology Co., Ltd. (Lot No. 110324200104031242, Beijing China), weighed almost 180 g. The animal experimental scheme is briefly drawn in Supplementary Figure S1. The SD rats were accommodated in the environment with 50 ± 10% humidity, 25 ± 1°C temperature, and 12-h light/dark cycles from 6 a.m. to 6 p.m. Food and water were provided *ad libitum*. A total of 72 rats experienced a 1-week adaption before the experiments began.

The acute MI model was established by ligating the LADCA according to a previous report (Wang et al., 2020). In brief, the pericardium was opened by anterior thoracotomy. Subsequently, the heart was immediately exteriorized, and the LADCA was ligated at the site at almost a 2 mm distance from its origin by a 5-0 polypropylene suture. During the experiment, 60 rats experienced LADCA ligation operation and 53 rats survived 24 h after the surgery. After the echocardiography evaluation, the LADCA of 48 rats was successfully ligated with EF values ranging from 36 to 50% (Supplementary Figure S2) and randomly divided into an MI group (*n* = 12), Met group (*n* = 12), NGR1-L group (*n* = 12), and NGR1-H group (*n* = 12) which received intragastric administration of physiological saline, 10 mg/kg/d Met, and 30 mg/kg/d and 60 mg/kg/d NGR1. A total of 12 rats which were subjected to anterior thoracotomy and the heart was threaded through without LADCA ligation and were assigned to the sham group and only received physiological saline. The animal experiment lasted 20 days.

### 2.4 Echocardiographic Assay

To investigate the echocardiographic changes after dosage, the SD rats were anesthetized with a mixture of O_2_/N_2_O (1:2) added with 3.0% isoflurane at 0.2 L/min, and the transthoracic echocardiographic studies were carried out by using a VINNO 6VET/6LAB ultrasound system (Mindary-BioMedical Electronics Co., Ltd., Shenzhen, China) equipped with an 18-MHz probe. The left ventricular (LV) end-systolic diameter, LV end-diastolic diameter, anterior wall systolic thickness, anterior wall diastolic thickness, posterior wall systolic thickness, and posterior wall diastolic thickness were acquired from the two-dimensional and M-mode echocardiograms. According to the aforementioned values, the LV ejection fraction (EF), fractional shortening (FS), and stroke volume (SV) were calculated. The echocardiography assays were conducted by a professional operator who was blinded to the animal grouping.

### 2.5 Sample Collection

The whole blood samples were drawn from the aorta abdominals of anesthetized rats. The serum samples were separated from whole blood samples after being settled at 25°C for 30 min and stored in a −80°C freezer. After heart perfusion by phosphate-buffered saline (PBS), the heart tissues were promptly split from the SD rats. Some heart tissues were applied for pathological assay, and others were stored at −80°C for the following tests.

### 2.6 Myocardial Infarction Assessment

After 24 h of refrigeration, the frozen heart was split into five thick short-axis sections (almost 1.5 mm per slice) along the apex to the base of the heart. These sections were first immersed in 1% 2,3,5-triphenyl tetrazolium chloride (TTC) solution for 30 min at 37°C, and then the sections were fixed with 4% paraformaldehyde for 1 h. The sliced images were captured by using a light microscope CKX41 (Olympus, Japan), and the infarction size was calculated as follows: infarction size (%) = LV infarction area/whole LV area, by the ImageJ software (NIH, MD, United States).

### 2.7 Pathological Histology and TUNEL Staining

The fresh heart tissues were briefly washed with PBS and then drained on a paper towel. The clean heart tissues were fixed with 10% formalin for 24 h at room temperature. Subsequently, the heart samples were embedded in paraffin and sectioned into 3 μm. The paraffin sections were subjected to regular hematoxylin & eosin staining (H & E) and Masson’s staining. TUNEL staining was performed according to the manufacturer’s instructions. After staining, the photographs were acquired by using a light microscope CKX41 (Olympus, Japan). The data of collagen contents, cross-section areas, and TUNEL-positive cardiomyocytes were analyzed using the ImageJ software.

### 2.8 Biochemical Indicators and Inflammatory Cytokines

The contents of LDH, CK-MB, α-HBDH, and FFA in the serum were tested by using commercial kits. The inflammatory cytokines IL-6, NF-κB, and TNF-α in the serum samples were also detected by using the commercial ELISA kits according to the manufacturer’s protocols. The absorbance values were detected by using a SPARK microplate reader (TECAN, Switzerland). The contents of biochemical indicators and inflammatory cytokines were calculated in accordance with the manufacturer’s instructions.

### 2.9 Proteomics Analysis

#### 2.9.1 Protein Sample Preparation

The protein extractions were performed by first pulverizing the aortas in liquid nitrogen and lysing the fragments in ice-cold lysis buffer (8 mol/L urea, 1% protease inhibitor cocktail). The homogenates were centrifuged at 15,000 rpm, 4°C for 10 min, and the supernatants were quantified by using a bicinchoninic acid (BCA) kit in accordance with the manufacturer’s instructions (Beyotime Biotechnology, Shanghai, China). Equivalent protein solutions from different groups were digested by trypsin, and the peptides were labeled using a TMT kit according to the manufacturer’s instructions (Thermo Scientific, United States).

#### 2.9.2 LC-MS Conditions for Proteomics Analysis

The LC-MS assays were carried out by PTM BIO (Hangzhou, China), and the detailed procedures were as follows. The tryptic peptides were fractionated using an Agilent 300Extend-C18 column (4.6 × 250 mm2, 5 µm) with a gradient of 8–32% acetonitrile (pH 9.0) over 60 min into 60 fractions. The fractionated peptides were dried and then redissolved in 0.1% formic acid for being loaded onto a homemade reversed-phase analytical column (150 mm × 75 µm) in an EASY-nLC 1000 UPLC system. The optimal mobile phase consisted of a linear gradient system of (A) 0.1% formic acid and (B) 0.1% formic acid in 98% acetonitrile: 0–26 min, B 6–23%; 26–34 min, B 23–35%; 34–37 min, B 35–80%; and 37–40 min, B 80%. The flow rate was 0.4 μl/min.

MS spectra were acquired by tandem mass spectrometry in Q ExactiveTM plus (Thermo). The optimal conditions were as follows: the electrospray voltage was 2.0 kV; the mass range for the full scan was set at 350–1800 m/z; the resolution of intact peptides was 70,000; one MS scan was followed by 20 MS/MS scans with 15 s dynamic exclusion; and the fixed first mass was set as 100 m/z. The standard for identifying differentially expressed proteins was set to be greater than 1.20-fold change, *p* < 0.05 (Student’s t-test, assuming unequal variance) and used for comparing proteins in the model group with proteins in the control groups.

Finally, gene ontology (GO, RRID: SCR_007,691) assignments were applied to classify proteins, and enriched functions were annotated with *p*-values corrected by FDR ≤0.05.

The mass spectrometry proteomics data have been deposited to the ProteomeXchange Consortium *via* the PRIDE partner repository with the dataset identifier PXD032814.

### 2.10 Target Prediction

The three-dimensional (3D) structures in the mol.2 format of NGR1 were drawn by ChemBio3D Ultra 14.0 software (PerkinElmer Inc., San Diego, CA, United States). To predict the targets, the 3D NGR1 were submitted to the Pharm Mapper Database (http://www.lilab-ecust.cn/pharmmapper) for reverse docking. The identified targets were then inputted into the Kyoto Encyclopedia of Genes and Genomes (KEGG) (http://www.kegg.jp/), GO, and the String 11.0 (https://string-db.org/) databases for protein functional enrichment and protein–protein interaction (PPI) prediction.

### 2.11 Western Blot Analysis

The protein extraction procedures were similar to the method described in 2.9.1. The equal fractions of proteins were separated by SDS-PAGE (10%), and then the separated proteins were transferred from SDS-PAGE gel onto PVDF membranes. Subsequently, the PVDF membranes were blocked by 5% skim milk at 37°C for 1 h and then incubated with primary antibodies against COX2 (1:1,000), CPT2 (1:1,000), CD36 (1:1,000), ACADVL (1:1,000), and GAPDH (1:2000) at 4°C overnight. The PVDF membranes were rinsed with PBST thoroughly and then incubated with HRP-conjugated secondary antibodies at room temperature for 1 h. After incubating with chemiluminescent HRP substrates, the protein bands were photographed by a Bio-Rad ChemiDoc™ MP Imaging System (Bio-Rad, United States). The relative intensities of proteins to GAPDH were calculated by ImageJ software.

### 2.12 *Conventional* MD Simulations

#### 2.12.1 System Preparation

The crystal structures of AKT (PDB: 4ekl) and mTOR-mLST8 (PDB: 4JT6) were downloaded from the Protein Data Bank (http://www. Rcsb. org/pdb). The crystal structures were prepared by AutoDock 4.2 software (Olson Laboratory, La Jolla, CA, United States). The binding mode construction of AKT and mTOR was performed by ZDOCK 3.0.2, while the ternary complex of AKT, mTOR, and NGR1 was assayed by Vina 1.1.2 software. Pymol software 1.8 (Schrödinge, Inc.) was applied to show structures and draw pictures, and Amber 18 software package was adopted to conduct the calculations.

#### 2.12.2 Conventional MD Simulations

The conventional MD simulations were carried out according to a previous report (Wu et al., 2018). Briefly, after the calculation of NGR1 charges by Gaussian 09 software, the optimized crystal structures of AKT and mTOR-mLST8 were applied as the initial structures for the conventional MD simulations. Molecular mechanics parameters from the GAFF2 force field and ff14SB force field were, respectively, assigned to NGR1 and proteins. The structures were solvated in a rectangular box of TIP3P water, with an extending range of 10 Å. For neutralization, a moderate amount of sodium and helium ions was added. At first, the water molecules were minimized by 2,500 steps of a conjugate gradient. Then, the TIP3P box was generally heated at a constant volume from 0 to 298.15 K over a coupling time of 100 ps. Next, the MD equilibrations were conducted for equilibrating the system. At last, a 50-ns production run in the *NPT* ensemble was performed. During the MD assay, the direct space interaction was calculated by the particle mesh Ewald method with a long-range electrostatic interaction (cutoff = 10.0 Å). All bonds involving hydrogen atoms were constrained with the SHAKE algorithm, with an integration time step of 2 fs.

### 2.13 Statistical Analysis

All data were reported as the mean value ±standard deviation (SD). For multiple comparisons, significant differences were conducted by one-way analysis of variance (ANOVA), and for single comparisons, significant differences were performed by Student’s *t*-test, using GraphPad (GraphPad software, version 5.0, San Diego, CA, United States). A *p* value < 0.05 was regarded as statistically significant. For the analysis of echocardiographic examinations, pathological indexes, and biochemical indicators, six rats were randomly selected in every analysis.

## 3 Results

### 3.1 Notoginsenoside R1 Ameliorated MI-Induced LV Dysfunction

**FIGURE 1:**
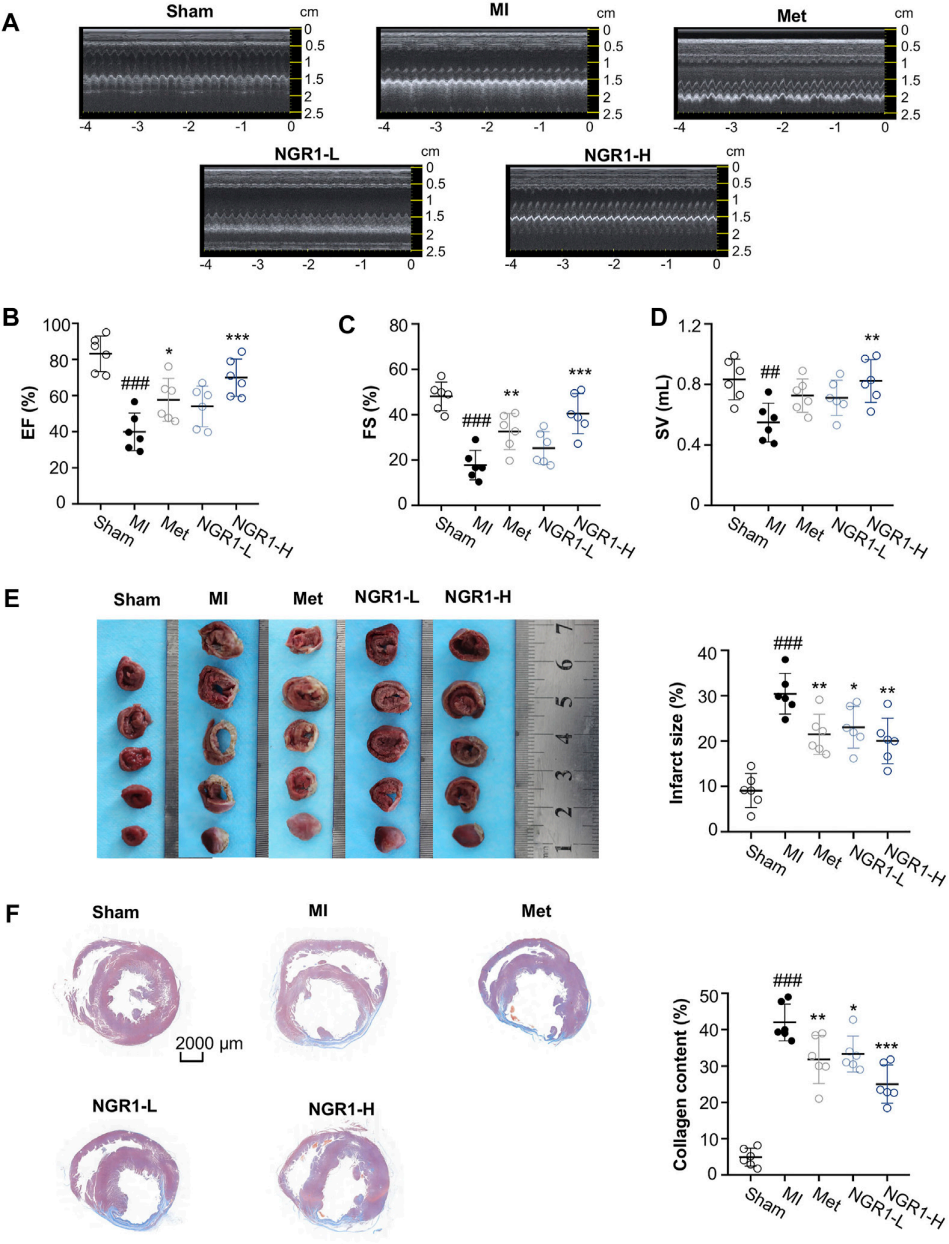
NGR1 alleviates LADCA ligation–induced ventricular dysfunctions, myocardial infarction, and fibrosis. **(A)** Representative electrocardiogram and **(B)** values of EF (left ventricular ejection fraction), **(C)** values of FS (left ventricular fraction shortening), and **(D)** values of SV (stroke volume). **(E)** Representative photos of rat heart slices by TTC staining and quantitative analysis of infarct size. **(F)** Representative images of rat heart slices by Masson’s staining and quantitative analysis of collagen content. Scale bar = 2000 μm. Values expressed as the mean ± SD. ^*^ *p* < 0.05, ^**^ *p* < 0.01, and ^***^ *p* < 0.001 vs. the MI group; ^#^ *p* < 0.05, ^##^ *p* < 0.01, and ^####^ *p* < 0.001 vs. the sham group (*n* = 6 for sham, MI, Met, NGR1-L, and NGR1-H groups).

In the current study, LADCA ligation was applied to establish an MI model in SD rats. The results of the transthoracic echocardiography assay demonstrated that the myocardial echo obviously weakened and the LV wall markedly thinned after ligating the LADCA in the MI group (Figure 1A). The Met and NGR1-H significantly intensified the myocardial echo and thickened the LV wall, while NGR1-L slightly improved the myocardial echo and LV wall thickness (Figure 1A). Compared with the sham group, three indicators of the LV systolic function, EF, FS, and SV, declined significantly in the MI group (Figures 1B–D). Met obviously increased the levels of EF and FS. In addition, NGR1-L slightly augmented the levels of the three indicators, while NGR1-H effectively increased the levels of EF, FS, and SV, which reflected that NGR1 exerted dose-dependent improvement of LV systolic function after LADCA ligation (Figures 1B–D).

### 3.2 Notoginsenoside R1 Improved MI-Induced Myocardial Infarction

To evaluate the improvement effect of NGR1 on myocardial infarction, TTC staining was carried out. The normal tissues were stained dark red by TTC, but the infarcted myocardial tissues could not be stained by TTC, thus appearing white. The TTC staining results revealed that LADCA ligation remarkably increased the infarct size of heart tissues in MI rats, while Met and NGR1 substantially attenuated myocardial infarction (Figure 1E).

### 3.3 Notoginsenoside R1 Attenuated MI-Induced Histopathological Lesions

To assess the improvement of NGR1 on myocardial fibrosis, Masson’s staining was carried out and the representative photomicrographs are shown in Figure 1F. In Masson’s staining, the collagen fiber was stained blue, and the muscle fiber was stained red. As shown in Figure 1F, excess collagen fibers accumulated in the lesion sites of the hearts of MI rats, while Met effectively improved myocardial fibrosis. NGR1-L attenuated collagen fiber accumulation in cardiac tissues, and NGR1-H also exerted an improvement effect which was better than that of NGR1-L (Figure 1F).

**FIGURE 2:**
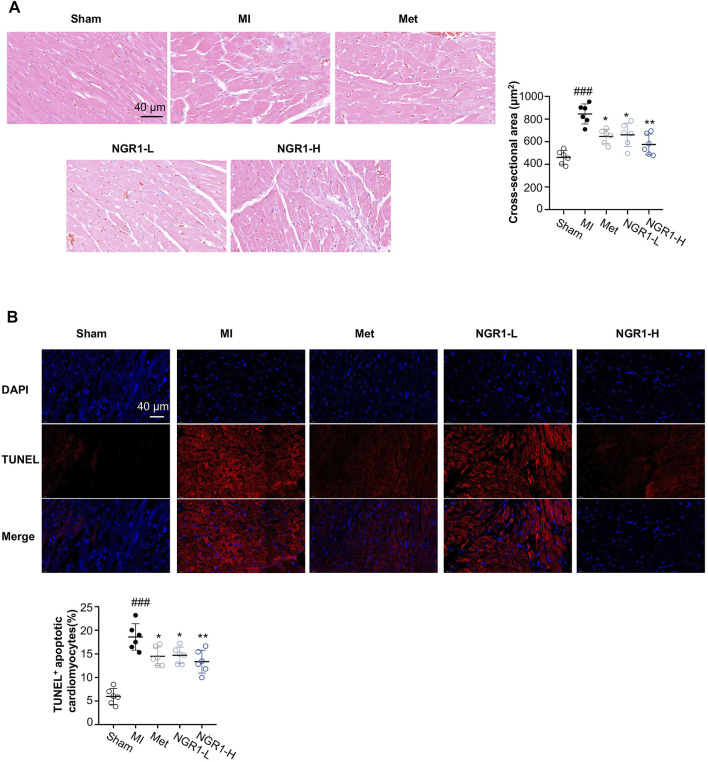
Representative images of histopathology and TUNEL staining. **(A)** Representative photomicrographs of cardiac tissue sections with H & E staining and quantitative analysis of the cross-sectional area. Scale bar = 40 μm. **(B)** TUNEL assay of apoptotic cardiomyocytes. Scale bar = 40 μm. Values expressed as the mean ± SD. ^*^ *p* < 0.05, ^**^ *p* < 0.01, and ^***^ *p* < 0.001 vs. the MI group; ^#^ *p* < 0.05, ^##^ *p* < 0.01, and ^####^ *p* < 0.001 vs. the sham group (*n* = 6 for sham, MI, Met, NGR1-L, and NGR1-H groups).

From the H & E staining shown in Figure 2A, swollen cardiomyocytes, infiltrated leukocytes, dilated intercellular gap, and disordered myocardial structure were observed in cardiac tissues from MI rats, while NGR1 administration ameliorated these pathological changes. To investigate the amelioration effect of NGR1 on myocardial hypertrophy, the cross-sectional areas were calculated from H & E–stained images, and the results indicated that NGR1-L and NGR1-H both substantially decreased the MI-induced cross-sectional area increment, which revealed that NGR1 relieved MI-induced myocardial hypertrophy (Figure 2A).

### 3.4 Notoginsenoside R1 Improved MI-Induced Myocardial Apoptosis

To quantify the amounts of apoptotic cardiomyocytes, TUNEL staining was performed. The TUNEL assay indicated that obvious red fluorescence was observed in the cardiac tissues of MI rats, which revealed that excessive myocardial apoptosis occurred in MI rats (Figure 2B). However, Met, NGR1-L, and NGR1-H effectively reduced the amounts of TUNEL-positive cells, thus relieving excessive myocardial apoptosis (Figure 2B).

### 3.5 Notoginsenoside R1 Suppressed Cardiac Markers and Inflammatory Cytokine Accumulation

**FIGURE 3:**
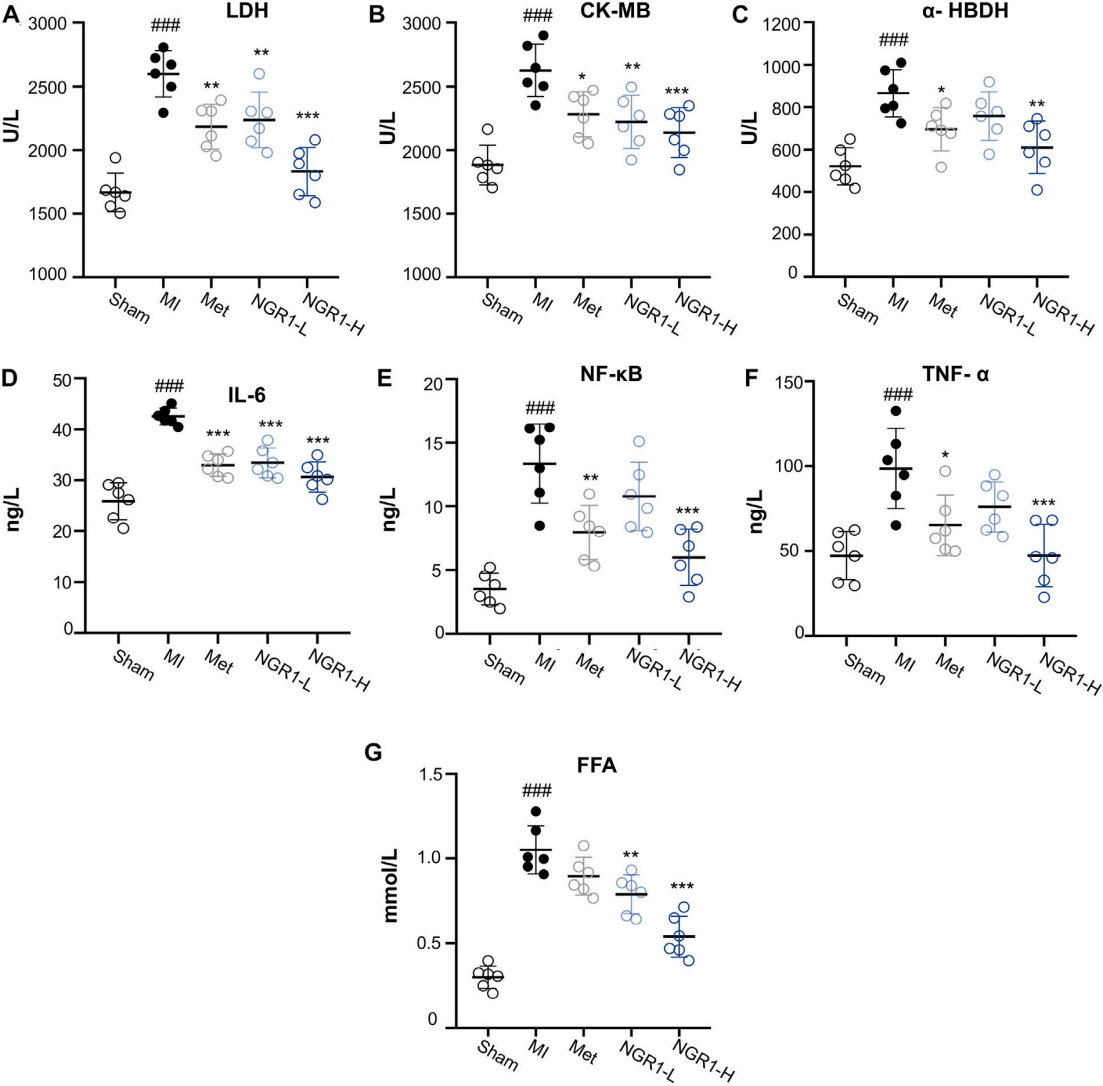
Effects of NGR1 on serum biochemical indicators in LADCA-ligated rats. **(A)** LDH; **(B)** CK-MB; **(C)** α-HBDH; **(D)** IL-6; **(E)** NF-κB; **(F)** TNF-α; and **(G)** FFA. Values expressed as the mean ± SD. ^*^ *p* < 0.05, ^**^ *p* < 0.01, and ^***^ *p* < 0.001 vs. the MI group; ^#^ *p* < 0.05, ^##^ *p* < 0.01, and ^####^ *p* < 0.001 vs. the sham group (*n* = 6 for sham, MI, Met, NGR1-L, and NGR1-H groups).

Specific myocardial injury markers, LDH, CK-MB, and α-HBDH, accumulated significantly in the serum of MI rats (Figures 3A–C). However, Met and NGR1-H administration effectively attenuated the accumulation of LDH, CK-MB, and α-HBDH in the serum, while NGR1-L obviously reduced the levels of LDH and CK-MB and slightly regulated the α-HBDH level in the serum, which suggested that NGR1 exerts dose-dependent myocardial protection (Figures 3A–C).

The serum contents of inflammatory cytokines, IL-6, NF-κB, and TNF-α, increased abundantly in MI rats, indicating that excessive inflammation stress occurred in MI rats (Figures 3D–F). NGR1-H efficiently suppressed the increase of IL-6, NF-κB, and TNF-α in the serum, with a better effect than that of Met. NGR1-L markedly relieved IL-6 accumulation and mildly suppressed the secretion of NF-κB and TNF-α (Figures 3D–F). The serum levels of FFA were extensively increased in MI rats, while NGR1-L and NGR1-H declined the FFA increase and Met exerted a slight effect, suggesting that NGR1 exerts a better effect on improving serum FFA accumulation than Met (Figure 3G).

### 3.6 Network Pharmacology Study Indicated That Notoginsenoside R1 Regulated Vasculature Development and Lipid Metabolism

**FIGURE 4:**
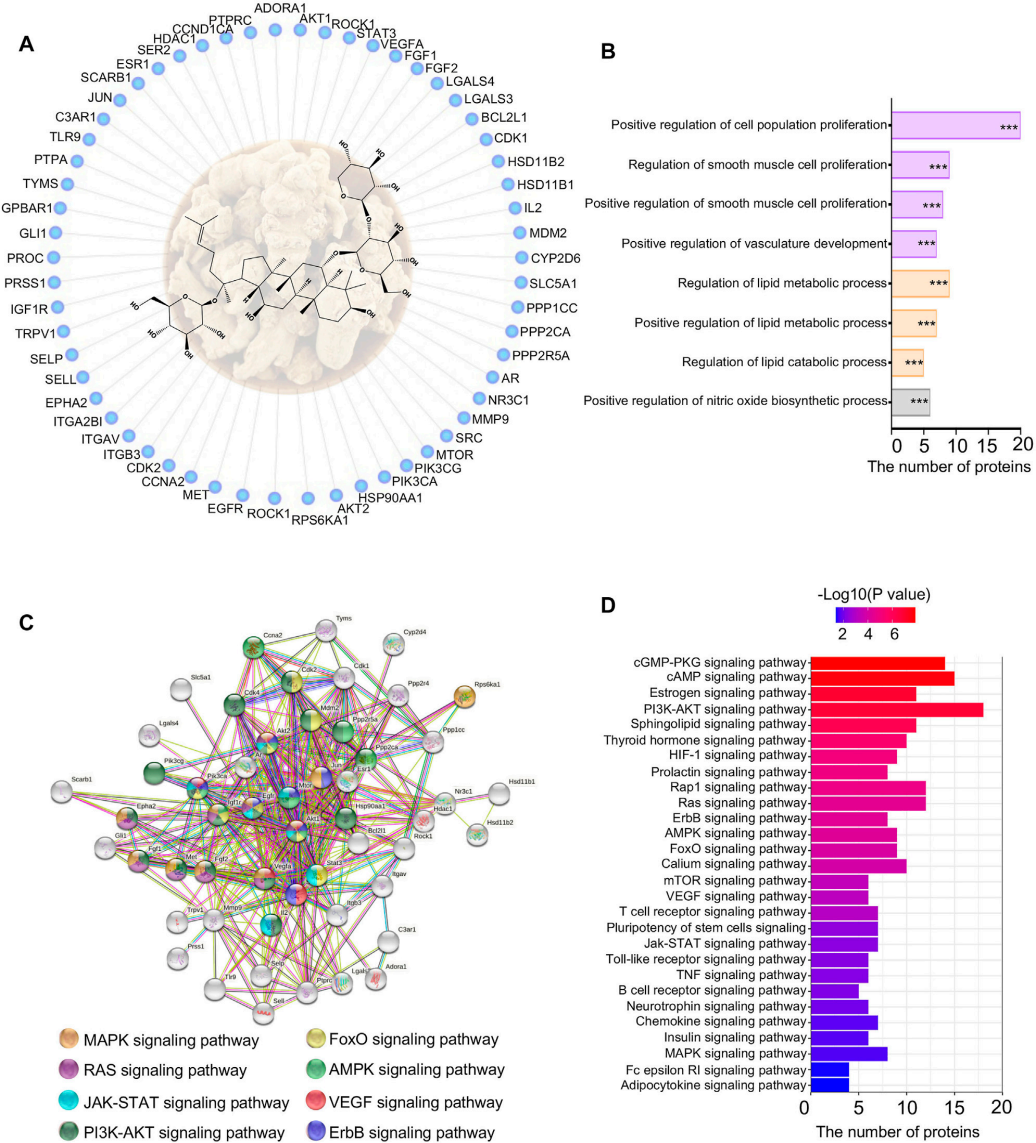
Network pharmacology of NGR1. **(A)** Reverse-docked targets of NGR1; **(B)** Functional annotation of reverse-docked targets. These reverse-docked targets were annotated by GO terms. The pathways were evaluated by a false-positive rate, ^*^ *F* < 0.05, ^**^ *F* < 0.01, and ^***^ *F* < 0.001. **(C)** Functional target association network by String analysis. **(D)** Functional annotation of reverse-docked targets by KEGG terms.

To identify potential mechanisms of NGR1 improving MI, a network pharmacology study was performed to predict targets of NGR1. Almost 62 targets were identified through reverse-docking, and the functional enrichment analysis by GO revealed that those targets are mainly enriched in smooth muscle cell proliferation, vasculature development, and lipid metabolism signaling (Figures 4A,B). The results of the PPI analysis suggested that these 62 targets were primarily involved in the cGMP-PKG pathway, cAMP pathway, and PI3K-AKT pathway (Figures 4C,D). Among these pathways, the PI3K-AKT pathway embodied extraordinary functional dependence, including 18 potential targets with -log (*p* value) of more than 6 (Figure 4D).

### 3.7 Proteome Profiling Revealed That Notoginsenoside R1 Regulated Metabolic Pathways

**FIGURE 5:**
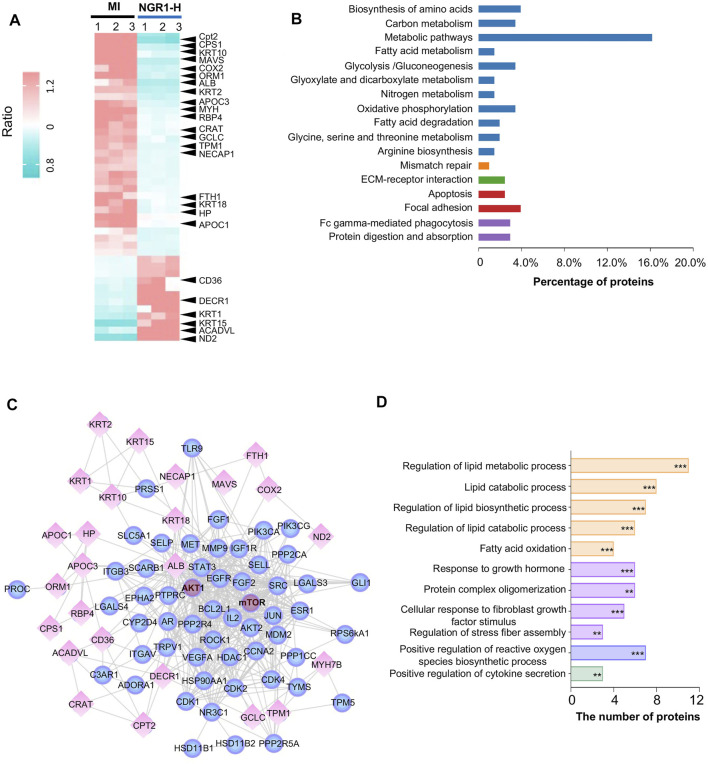
Myocardial proteomics combined with network pharmacology revealed that NGR1 improved lipid metabolism in the ischemic myocardium. **(A)** Heatmap of differential proteins between the NGR1-H group and the MI group. **(B)** Functional annotation of differential proteins. **(C)** Lipid metabolism–associated network including reverse-docked targets and differential proteins. Spheres represent reverse-docked targets and diamonds represent differential proteins. **(D)** Functional annotation of proteins in the lipid metabolism–associated network. These proteins in the lipid metabolism–associated network were annotated by GO terms. The pathways were evaluated by a false-positive rate, ^*^ *F* < 0.05, ^**^ *F* < 0.01, and ^***^ *F* < 0.001.

Through the proteomics analysis, a total of 96 differential proteins between the MI group and sham group were identified, including 49 downregulated proteins and 47 upregulated proteins (Supplementary Figures S3A,B). Among these 96 differential proteins between the MI and sham groups, we filtered out 44 significantly differential proteins between the NGR1-H and MI groups (Supplementary Figures S4A,B). Among these 44 differential proteins, 32 differential proteins were significantly upregulated between the MI and sham groups and were effectively decreased after NGR1-H administration. The other 12 differential proteins were downregulated after LADCA ligation while they were increased by NGR1-H treatment (Supplementary Figures S4A,B, Figure 5A). These 44 differential proteins improved by NGR1-H were enriched in metabolic progresses and energy supply processes (Figure 5B).

### 3.8 AKT and mTOR Were the Pivotal Targets of Notoginsenoside R1

To predict the targets of NGR1 in ameliorating MI, we performed biological function enrichment and PPI analysis, combining the potential targets in network pharmacology and the 44 differential proteins in proteomics, and the network of potential targets and critical differential proteins were established (Figure 5C). This network is mainly involved in lipid metabolism and second in the fibrosis process, which suggested that NGR1 plays a critical role in improving lipid metabolism and alleviating the fibrosis process in ischemic heart (Figure 5D). In this network, every edge represented an association between two proteins, and thus, a protein with more edges was a core target involved in more pathways. Moreover, guiding by the PI3K-AKT pathway in *3.6*, AKT and mTOR were identified at the core sites of this network, which indicated that AKT and mTOR were pivotal targets of NGR1.

### 3.9 Verification of Vital Proteins in Improving Lipid Metabolism by Notoginsenoside R1

**FIGURE 6:**
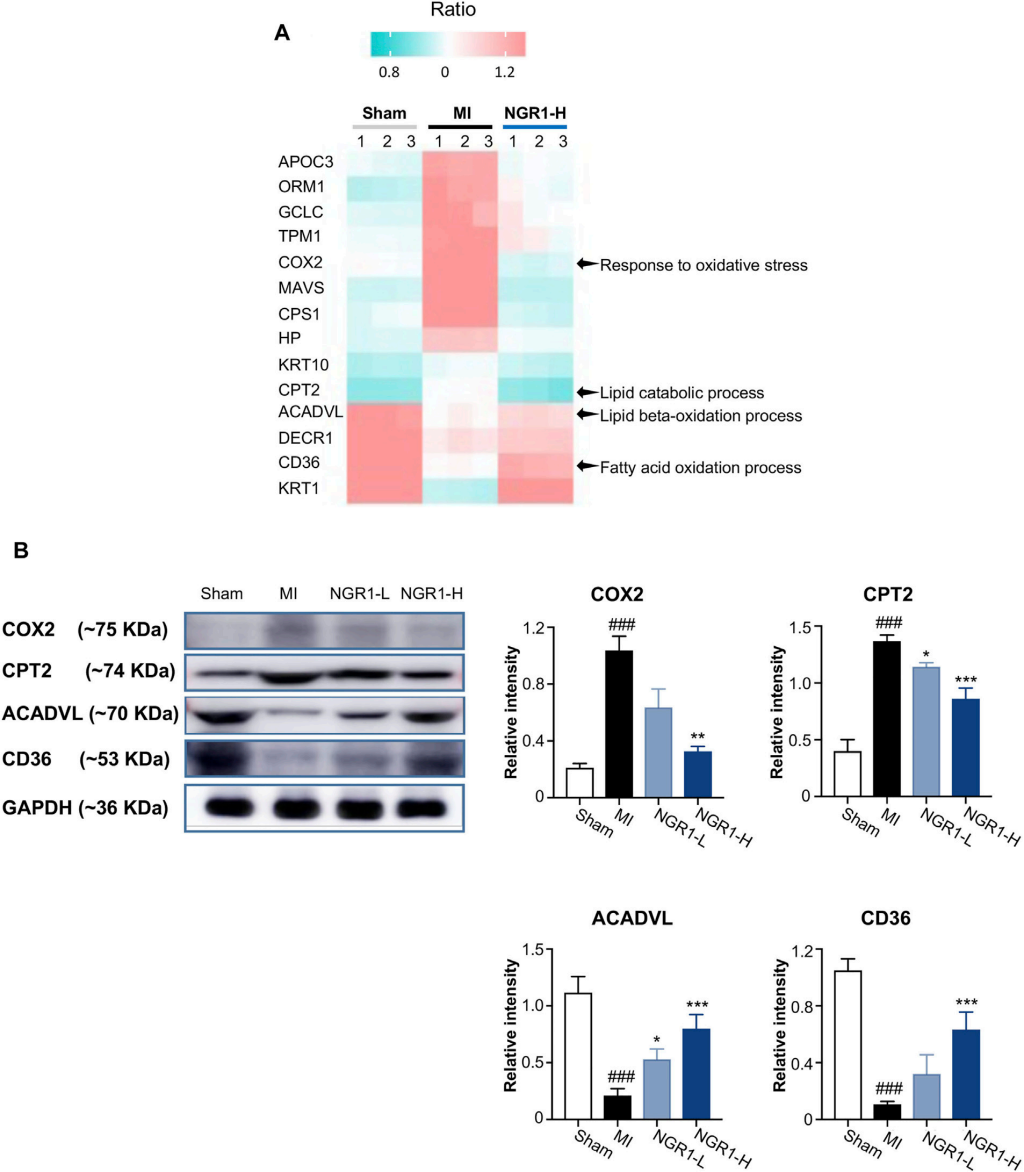
Validation of key differential protein expression in the lipid metabolism–associated network. **(A)** Heatmap of differential proteins in the lipid metabolism–associated network. **(B)** Expression of COX2, CPT2, ACADVL, and CD36 by Western blots. Values expressed as the mean ± SD. ^*^ *p* < 0.05, ^**^ *p* < 0.01, and ^***^ *p* < 0.001 vs. the MI group; ^#^ *p* < 0.05, ^##^ *p* < 0.01, and ^####^ *p* < 0.001 vs. the sham group (*n* = 3 for sham, MI, NGR1-L, and NGR1-H groups).

The critical proteins regulated by NGR1 for improving lipid metabolism were identified by the combined network in *3.8* and the heatmap representing the expression of these critical proteins in myocardial proteomics is shown in Figure 6A. The expression levels of five functional proteins were verified by Western blots. Acyl-CoA dehydrogenase very long-chain (ACADVL) and carnitine palmitoyltransferase-2 (CPT2) are important β-oxidation enzymes in the mitochondria (Yamada and Taketani, 2019). The expression of ACADVL was significantly decreased in the MI group compared with the sham group, indicating the defects in fatty acid β-oxidation in myocardium mitochondria, while NGR1 attenuated the decrement of ACADVL. CPT2 exerts its functionality by simultaneously regulating fatty acid and glucose metabolism (Rufer et al., 2009). In our study, the expression of CPT2 was upregulated in the MI group compared with the sham group, which suggested that there were disorders between fatty acid β-oxidation and glycolysis. NGR1 administration effectively improved the abnormal increase of CPT2 in ischemic myocardium (Figure 6B). CD36, as a dynamic gatekeeper of fatty acids, markedly declined in the ischemic myocardium, suggesting subdued β-oxidation (Glatz et al., 2021). NGR1 significantly upregulated the expression of CD36 in the ischemic myocardium, which suggested that NGR1 partly restores β-oxidation (Figure 6B). The expression of cyclooxygenase (COX2), an important lipoxygenase, was upregulated in the ischemic myocardium, indicating enhanced lipid peroxidation (Clemente et al., 2020). However, NGR1 downregulated the expression of COX2, which might suggest that NGR1 restores lipid metabolism by suppressing lipid peroxidation (Figure 6B). Our results demonstrated that NGR1 improved the lipid metabolic disorder and defect of fatty acid β-oxidation in MI rats.

### 3.10 Notoginsenoside R1 Interfered the Protein–Protein Interaction of mTOR and AKT, Regulating the Lipid Metabolism

The activation of the PI3K/AKT/mTOR pathway results in enhancing lipid biosynthesis by upregulating the expression of SREBP1 and SREBP2 and increasing mitochondrial fatty acid β-oxidation (Aoki and Fujishita, 2017). mTOR exerts functionality by forming two heterotrimers mTORC1 and mTORC2 which both contain mTOR and mLST8 subunits. In our study, AKT and mTOR were both identified as the potential targets of NGR1, while these two proteins show distinct structures, actually. Therefore, we proposed that NGR1 might interfere with the interaction of AKT and mTOR.

**FIGURE 7:**
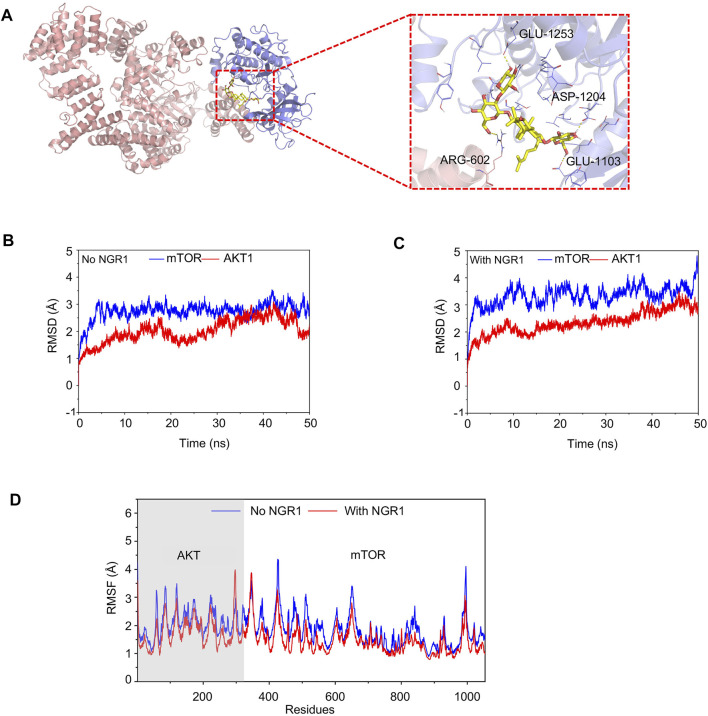
Conventional MD simulation for evaluating the binding affinity of mTOR and AKT with or without NGR1. **(A)** Overview structure of the mTOR-AKT complex with NGR1 and a zoom-in view of NGR1 and the surrounding amino acids. **(B)** Backbone (alpha-C, C, N atoms) RMSDs are shown as a function of time for mTOR and AKT without NGR1 during 50-ns molecular dynamics simulation. **(C)** Backbone (alpha-C, C, and N atoms) RMSDs are shown as a function of time for mTOR and AKT with NGR1 during 50-ns molecular dynamics simulation. **(D)** RMSF of mTOR and AKT residues in 50-ns molecular dynamics simulation.

To investigate the interference of NGR1 to mTOR and AKT interactions, the conventional MD simulation was carried out. In the AKT-mTOR complex, the first 341 amino acids belong to AKT, and the other amino acids belong to mTOR. In Figure 7A, NGR1 is bound into the cargo pocket of the ATP binding site in AKT and inserted into the binding site of mTOR and AKT by forming hydrogen bonds to AGR-602 of AKT and to ASP-1204, GLU-1103, and GLU-1253 of mTOR (Figure 7A).

The value of root-mean-square deviation (RMSD) represents the stability of backbone structures in the complex, and the greater the volatility, the more the instability. In this study, the backbone structure of AKT showed better stability in the AKT-NGR1-mTOR complex rather than in the AKT-mTOR complex (Figures 7B,C). The backbone structures of mTOR exhibited organized fluctuations around 2.97 Å in the AKT-mTOR complex and 3.42 Å in the AKT-NGR1-mTOR complex (Figures 7B,C), which indicated that the backbone structures of mTOR were stable in both complexes. The root-mean-square fluctuations (RMSF) manifested the stability of residues in AKT and mTOR, and the larger the fluctuation, the more instability. From Figure 7D, the residues of AKT and mTOR showed smaller fluctuations of RMSF in the AKT-NGR1-mTOR complex rather than that in the AKT-NGR1 complex, which suggested that molecules are more stable in a ternary complex than in a binary complex. Moreover, we also observed that only the local structures of the binding site with NGR1 showed larger fluctuations than that without NGR1; thus, NGR1 changed the local structures of AKT-mTOR.

## 4 Discussion

NGR1 is reported to be effective for cardiovascular protection, gastrointestinal protection, neuroprotection, and other biological activities, which contributes to the beneficial functions of *Radix notoginseng* (Ruan et al., 2010; Yang et al., 2014; Chan et al., 2019). However, NGR1 has a low oral bioavailability because of its large polarity, and it is mainly metabolized into ginsenosides Rg1, F1 and 20(s)-protopanaxatriol (PPT), and dehydrogenated PPT by intestinal bacteria (Li et al., 2014). To overcome the low bioavailability of NGR1, many alternatives had been attempted, such as co-administration with borneol, core-shell hybrid liposomal vesicle nanocarriers, poly (D,L-lactide-*co*-glycolide acid) nanoparticles, and bio-adhesive tablet, which markedly increased the bioavailability and activities of NGR1 (Zou et al., 2017; Xu et al., 2019; Liu et al., 2020). In our results, LADCA ligation disordered ventricular dysfunction, promoted fibrosis and apoptosis of the myocardium, and increased infarct size. However, NGR1 significantly improved the echocardiographic, tissue pathological, and myocardial biochemical perturbations in LADCA-ligated rats. Our results validated the protective effect of NGR1 on the ischemic heart.

In the acute ischemic myocardium, the principal energy supply shows a shift from fatty acid β-oxidation toward glycolysis, which leads to depressed energy efficiency and accumulated fatty acids (Qun Chen et al., 2018). Moreover, accelerated glycolysis and accumulated fatty acids result in the abnormal content of K^+^ and Ca^2+^, decreased pH, and cell rupture and death, which leads to more than a 10-fold increment in fatty acids released from the myocardium (Jeong et al., 2018; Horikoshi et al., 2019). Therefore, an increase in circulating fatty acids in acute myocardial ischemic patients has been observed (Feng et al., 2018). In our study, we also observed a significant increase of circulating free fatty acids in LADCA-ligated rats, while NGR1 effectively decreased the amounts of circulating fatty acids.

To uncover the potential pharmacological mechanism of NGR1 in protecting ischemic cardiac and alleviating circulating lipid accumulation, myocardial proteomics and network pharmacology were conducted. The results of myocardial proteomics of NGR1 highlighted the functions of regulating metabolism pathways including fatty acid oxidation, oxidative phosphorylation, glycolysis, and others, while the pharmacological network of NGR1 mainly focused on the regulations of smooth muscle cell proliferation and lipid metabolic progress. The results of myocardial proteomics combined with network pharmacology suggested that NGR1 improves ischemic heart diseases by regulating lipid metabolism and the AKT/mTOR pathway.

The mammalian target of rapamycin (mTOR) exists in two distinct multi-subunit complexes, mTORC1 and mTORC2, both sharing the namesake kinase, mTOR and its binding partner, mLST8 (Xizi Chen et al., 2018). mTORC2 can translocate to the plasma membrane and feel extracellular stimulations to activate mTORC1 by directly phosphorylating Ser473 of AKT (Martinez Calejman et al., 2020). The activated mTORC1 phosphorylates the bHLH leucine zipper transcription factor (TFEB) and upregulates the expression of PPARα and PPARrco-activatorα (PGC1α), thus accelerating fatty acid β-oxidation and lipid catabolism (Ricoult et al., 2016). In this study, conventional MD simulation indicated that NGR1 increased the stability of the mTOR- AKT complex, which promoted AKT/mTOR activation. Consistent with our observations, NGR1 activated the AKT/mTOR signaling pathway and increased the phosphorylation levels of AKT, both in high glucose–stimulated podocytes and in oxygen-glucose deprivation/reoxygenation-stimulated cortical neurons and unilateral carotid artery–ligated rats (Huang et al., 2017; Tu et al., 2018). The hyperactivation of the AKT pathway and the elevated CPT2 expression level were both observed in HMG-CoA reductase degradation protein 1 (HRD1)–deficient mice that showed high levels of fatty acid oxidation and suppressed lipogenesis (Wei et al., 2018). Furthermore, reduced AKT phosphorylation was accompanied by altered expression of CD36 and ACADVL in small heterodimer partner (SHP) overexpressed cardiac cells which showed obvious lipid accumulation and insulin resistance in cardiomyocytes (Rodríguez-Calvo et al., 2017). In our study, NGR1 activated the AKT/mTOR pathway, suppressed the expression of CPT2, and elevated CD36 and ACADVL expressions in ischemic cardiac cells, which was mutually verified with previous studies. Above all, our work demonstrated that NGR1 activated the AKT/mTOR pathway, thus increasing fatty acid β-oxidation and lipid catabolism and protecting ischemic myocardia against lipid overload–induced injury.

Although accurate methods of computing the affinity of biological targets with small molecules, such as conventional MD simulation, structure-based molecular docking, and quantitative structure-affinity relationships, have been and will have been reformed for drug discovery, the eutectic and the X-ray crystallographic analyses are still the most direct evidence for evaluating the binding state of a target protein and its ligands. However, only few crystal structures of the target protein and its ligands can be successfully grown because of the harsh terms. Therefore, lacking direct and effective evidence for assessing the binding state of a target protein and its ligands is still plaguing us.

## 5 Conclusion

In the present study, NGR1 ameliorated echocardiographic function, improved ventricular dysfunction, decreased infarct size, alleviated myocardial fibrosis, hypertrophy and apoptosis, and declined myocardial injury markers in LADCA-ligated rats, which suggested that NGR1 exerts significant cardio-protective effects against myocardial ischemia. Myocardial proteomics combined with network pharmacology revealed that NGR1 attenuated lipid accumulation and restored fatty acid β-oxidation by activating the AKT/mTOR pathway. Further conventional MD simulation uncovered that NGR1 increased the stability of the mTOR-AKT complex and promoted AKT/mTOR activation. In brief, NGR1 activated the AKT/mTOR pathway by stabilizing the mTOR-AKT complex and thus protected ischemic myocardia against lipid overload–induced injury. Our work also highlighted the lipid regulation effect of *Radix notoginseng* in cardio-protection. In addition, proteomics combined with network pharmacology and conventional MD simulation can be an efficient means for the profound mechanism evaluation of active natural product molecules.

## Data Availability

The datasets presented in this study can be found in online repositories. The names of the repository/repositories and accession number(s) can be found in the article/Supplementary Material.
