# Supplementary material for: Notoginsenoside R1 Regulates Ischemic Myocardial Lipid Metabolism by Activating the AKT/mTOR Signaling Pathway
Source: Front Pharmacol. 2022 Jun 22;13:905092. doi: 10.3389/fphar.2022.905092 (PMC9257227; doi:10.3389/fphar.2022.905092)

**Notoginsenoside R1 regulates ischemic myocardial lipid metabolism through activating AKT/mTOR signaling pathway**

Wei Lei^1,2,3^, Yiqi Yan^1,2,3^, Yaolei Ma^1,2,3^, Min Jiang^4^, Boli Zhang^1,2,3^, Han Zhang^1,2,3^*, Yuhong Li^1,2,3^*

*^1^ Key Laboratory of Pharmacology of Traditional Chinese Medical Formulae, Ministry of Education, Tianjin University of Traditional Chinese Medicine, 10 Poyanghu Road, Jinghai District,Tianjin 301617, China*

*^2^ State Key Laboratory of Component-based Chinese Medicine, Tianjin University of Traditional Chinese Medicine, Tianjin 301617, China*

*^3^ Institute of Traditional Chinese Medicine, Tianjin University of Traditional Chinese Medicine, Tianjin 301617, China*

*^4^ State Key Laboratory of Medicinal Chemical Biology, College of Pharmacy and Tianjin Key Laboratory of Molecular Drug Research, Nankai University, Tianjin 300353, China*

Running title: NGR1 protects ischemic cardiac by regulating lipid metabolism

*Corresponding author. Yuhong Li (professor)

Key Laboratory of Pharmacology of Traditional Chinese Medical Formulae, Ministry of Education, Tianjin University of Traditional Chinese Medicine, 10 Poyanghu Road, Jinghai District, Tianjin 301617, China.

Tel: +86 022 59596164; Fax: +86 022 59596164

E-mail: [liyuhong@tjutcm.edu.cn](mailto:liyuhong@tjutcm.edu.cn)

**Supplementary Figure 1.** The animal experimental flow graph

**
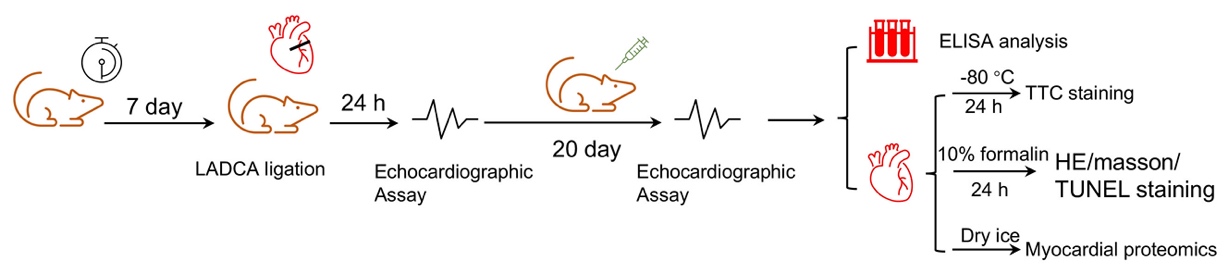
**

**Supplementary Figure 2.** EF values (left ventricular ejection fraction) of rats after LADCA ligating surgery in 24 h by cardiac electrocardiography.


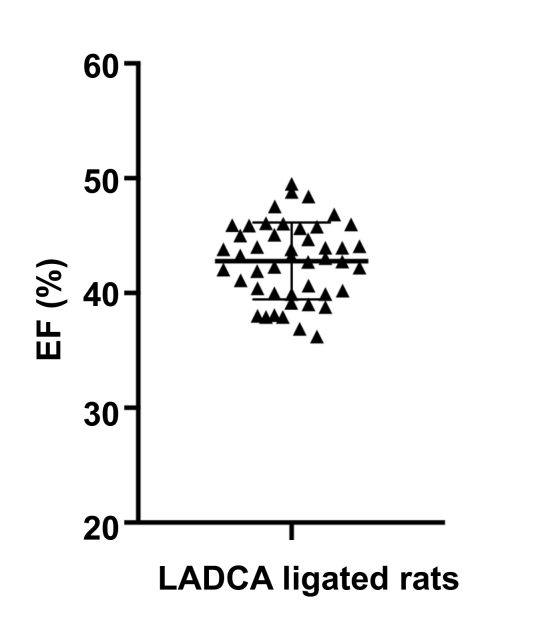


**Supplementary Figure 3.** (A) Heatmap of differentially expressed proteins between sham and MI groups (MI group vs. Sham group). (B) A volcano plot displaying log2 (fold change) and -log (p value) for all proteins identified by LC-MS in sham and MI groups. The red color marked up-regulated proteins and the blue color marked down-regulated proteins.


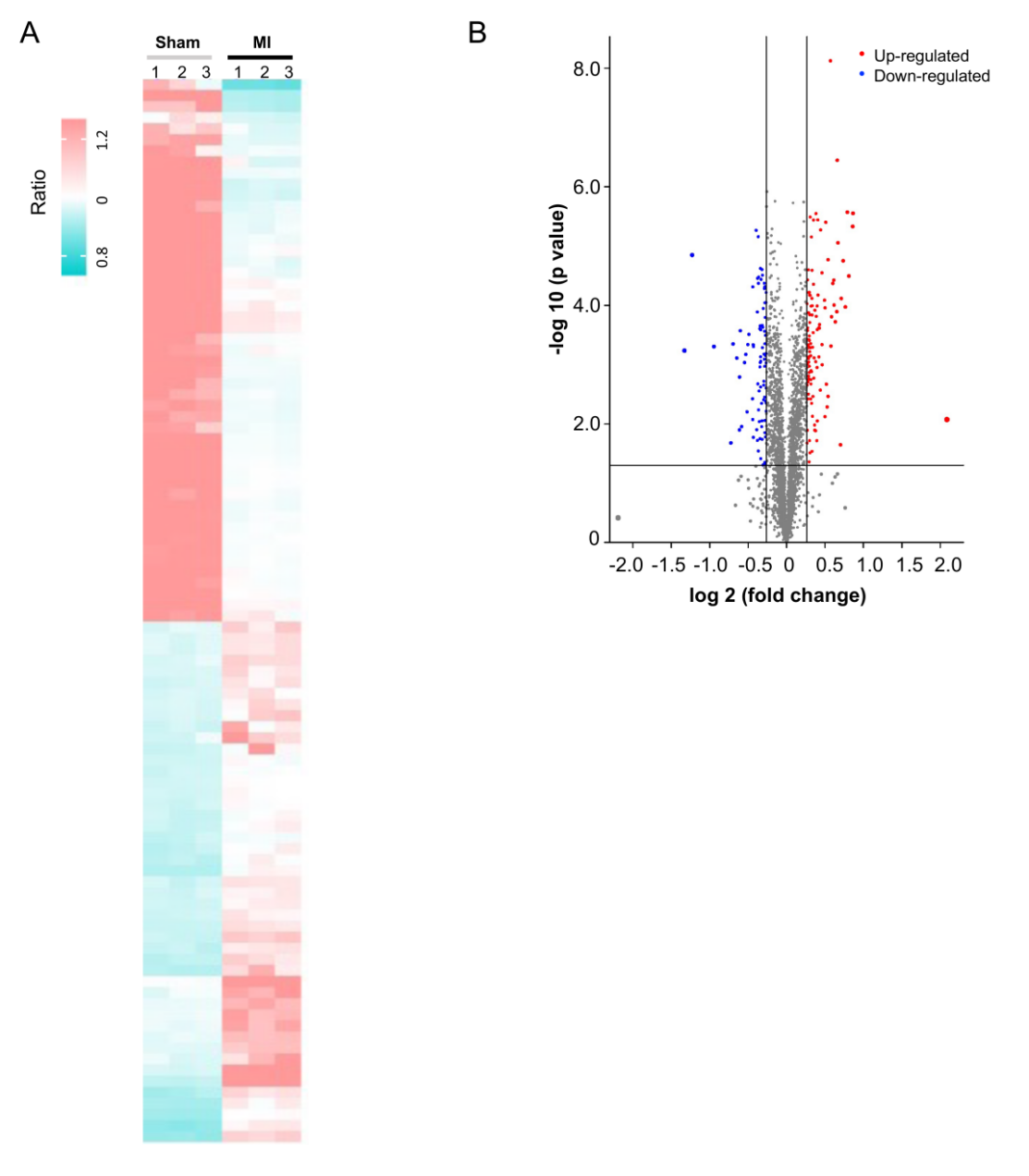


**Supplementary Figure 4.** (A) Heatmap of differentially expressed proteins between MI and NGR1-H groups (NGR1-H group vs. MI group). (B) A volcano plot displaying log2 (fold change) and -log (p value) for all proteins identified by LC-MS in MI and NGR1-H groups. The red color marked up-regulated proteins and the blue color marked down-regulated proteins.


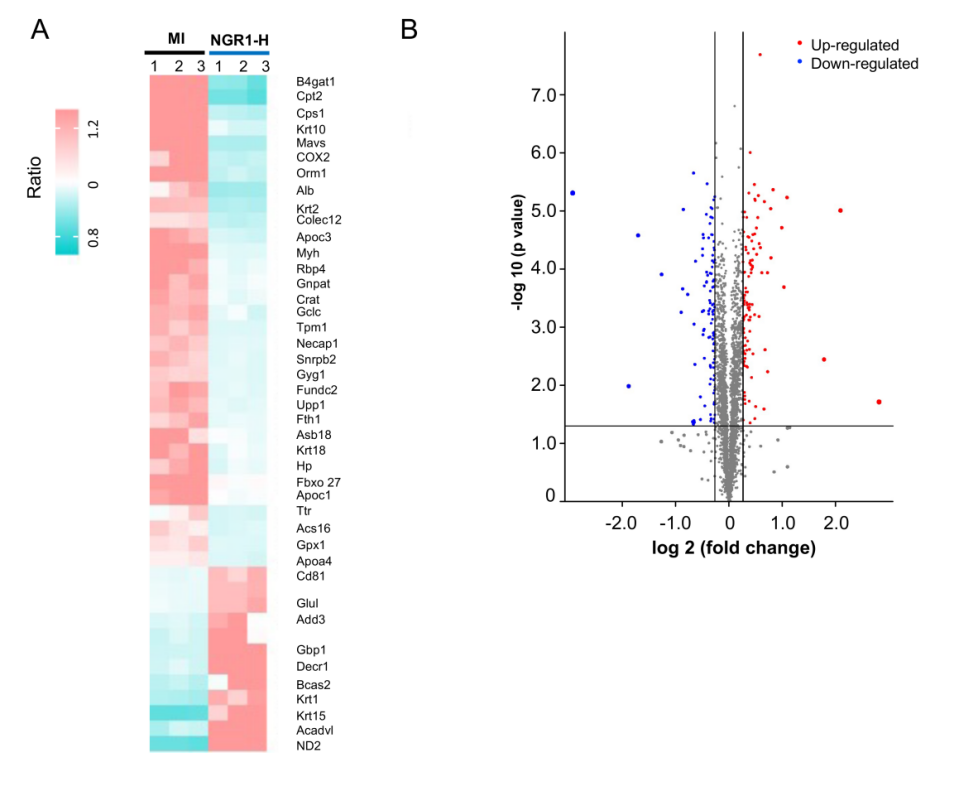


**Original Western Blots**


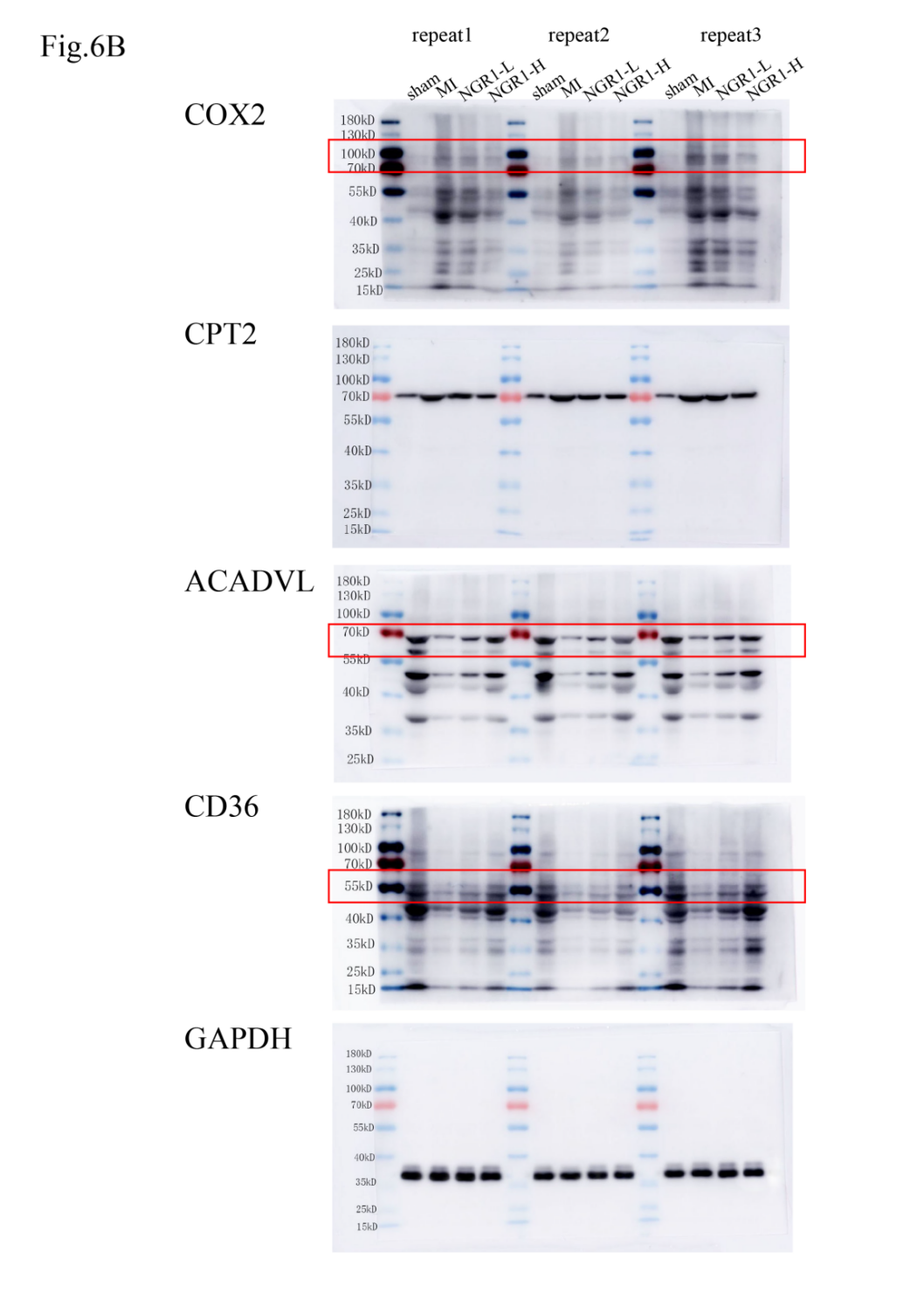

Supplement: Supplementary file 1 [file DataSheet1.docx]
